# Relationship between the frequency of electrocautery of Hunner lesions and changes in bladder capacity in patients with Hunner type interstitial cystitis

**DOI:** 10.1038/s41598-020-80589-3

**Published:** 2021-01-08

**Authors:** Yoshiyuki Akiyama, Masayoshi Zaitsu, Daiji Watanabe, Itsuki Yoshimura, Aya Niimi, Akira Nomiya, Yuta Yamada, Yusuke Sato, Masaki Nakamura, Taketo Kawai, Daisuke Yamada, Motofumi Suzuki, Haruki Kume, Yukio Homma

**Affiliations:** 1 Department of Urology, Graduate School of Medicine, The University of Tokyo, 7-3-1, Hongo, Bunkyo, Tokyo, Japan; 2 Department of Public Health, Dokkyo Medical University School of Medicine, Tochigi, Japan; 3 Depratment of Urology, Teikyo University School of Medicine, Tokyo, Japan; 4 Department of Urology, New Tokyo Hospital, MatsudoTokyo, Chiba Japan; 5 Department of Urology, National Center for Global Health and Medicine, Tokyo, Japan; 6 Japanese Red Cross Medical Center, Tokyo, Japan

**Keywords:** Urology, Bladder

## Abstract

Electrocautery is a promising treatment option for patients with Hunner type interstitial cystitis (HIC), but frequently requires multiple sessions due to recurrence of the lesions. In the present study, we assessed the relationship between the frequency of electrocautery of Hunner lesions and changes in maximum bladder capacity (MBC) at hydrodistension in a large cohort of 118 HIC patients. Three mixed-effect linear regression analyses were conducted for MBC against (1) the number of sessions; (2) the number of sessions and the time between each session and the first session; and (3) other relevant clinical parameters in addition to the Model (2). The mean number of sessions was 2.8 times. MBC decreased approximately 50 mL for each additional electrocautery session, but this loss was offset by 10 mL for each year the subsequent session was postponed. MBC of < 400 mL at the first session was a significant risk factor for MBC loss with further sessions. No other clinical parameters were associated with MBC over time. This study demonstrates a significant relationship between the frequency of electrocautery of Hunner lesions and MBC changes in HIC patients. Low MBC at the first session is a poor prognostic marker for MBC loss over multiple sessions.

## Introduction

Recently, Hunner type interstitial cystitis (HIC) has emerged as a distinct category of interstitial cystitis/bladder pain syndrome (IC/BPS)^1–3^. Hunner lesions, reddish mucosal lesions accompanied by abnormal capillary structures in the bladder, are a characteristic marker of HIC^4^. HIC is clinically and biologically distinct from BPS (other forms of IC/BPS)^5–7^. HIC patients have characteristic clinical features such as an older age of onset, more severe bladder-centric symptoms, and reduced bladder capacity compared with BPS patients^8,9^. Additionally, HIC is characterized by robust inflammatory changes, including epithelial denudation and frequent expansion of infiltrating B cells in the bladder^10,11^. Altered expression of genes associated with immune responses and infection processes have also been reported^5^.

Clinically, it is well known that HIC patients benefit from Hunner lesion-targeted therapies such as fulguration/resection of the lesions or direct injection of steroids at the lesion sites^12–14^. Electrocautery of Hunner lesions, which is usually performed with simultaneous bladder hydrodistension, is a promising surgical intervention for HIC patients. However, the effect is not long-lasting, and repeated sessions are frequently required^13,15^. There is therefore a growing concern that repeated sessions of electrocautery of Hunner lesions may affect bladder capacity, ultimately resulting in bladder contraction. Previous studies reported that multiple sessions of electrocautery of Hunner lesions, with or without hydrodistension, did not negatively affect bladder capacity over time^16–18^. However, those studies were conducted by a variety of methodology, including the varying number of study subjects and observation period, or different statistical methods and evaluation criteria (e.g., functional bladder capacity or bladder capacity at hydrodistension), which could have hindered dispelling this concern. In this study, using a large patient cohort and mixed-effect statistical models, we investigated the association between the frequency of electrocautery and maximum bladder capacity (MBC) at hydrodistension.

## Results

**Figure 1:**
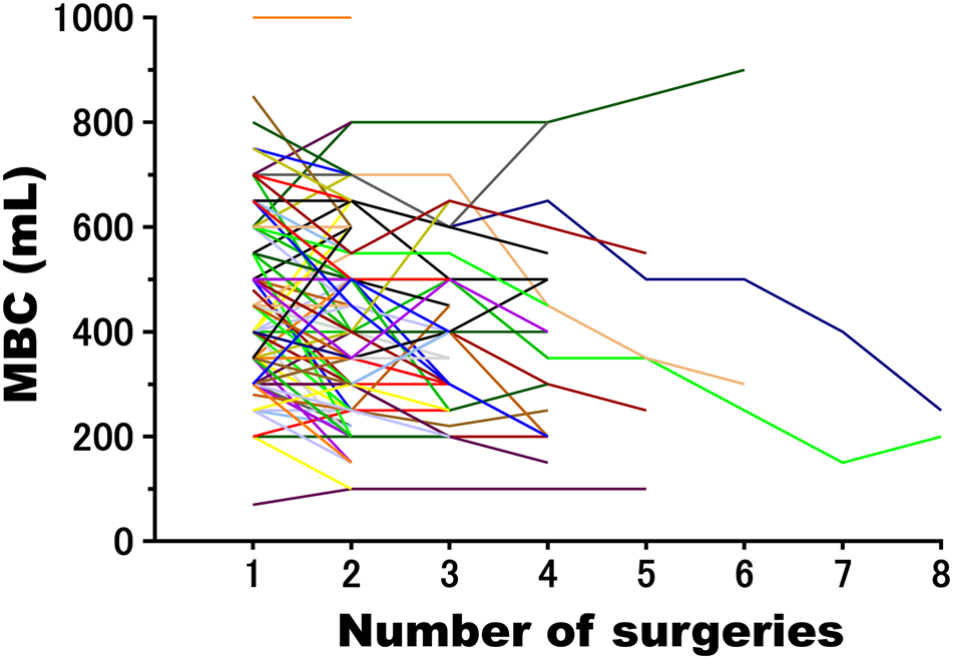
Spaghetti plot for MBC and the number of surgeries. Measurement of MBC over multiple sessions. Each colored line represents an individual patient.

**Figure 2:**
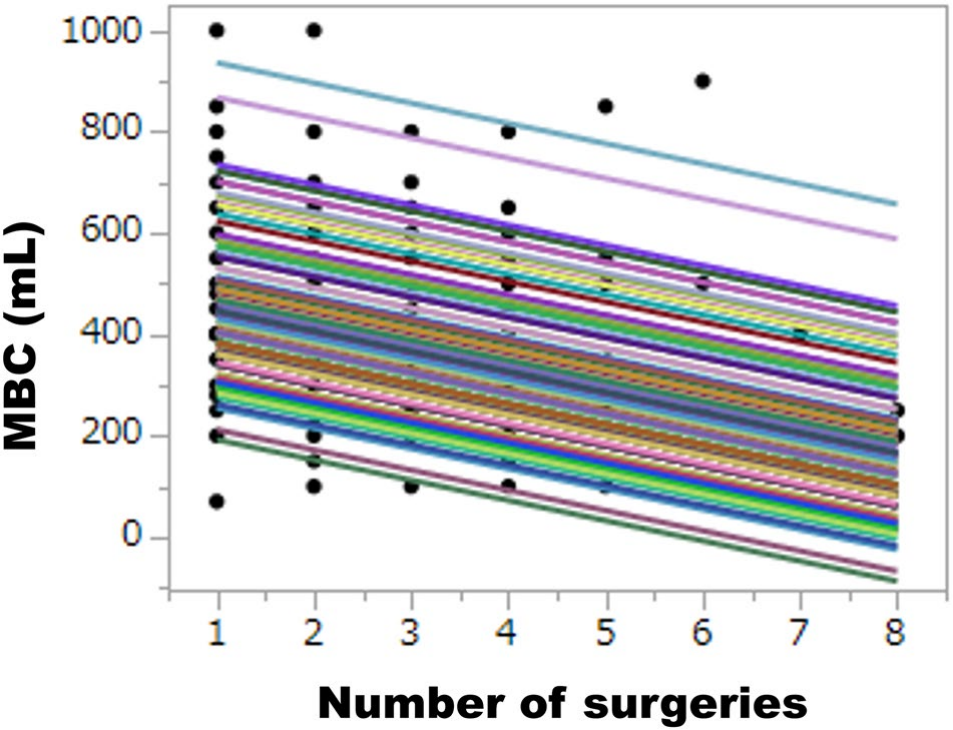
A mixed-effect regression model for MBC and the number of surgeries. The dots represent individual MBC measurements over time. Each colored line depicts the fitted regression line for an individual patient, with a fixed slope and random intercepts.

A total of 118 HIC patients (102 females and 16 males) met the inclusion criteria and were analyzed in this study (Table 1). The mean age at symptom onset, duration of illness (years from symptom onset to the first visit to our institution), and follow-up period (time from the first to the last session) were 62.8 years (range, 30–86 years), 3.2 years (0–23 years), and 38.5 months (5–134 months), respectively. The number of electrocautery sessions varied across patients, with a mean number of 2.7 ± 1.2 (range, 2–8; median, 2) (Fig. 1). A mixed-effect linear regression analysis for MBC against the number of sessions (Model 1, Fig. 2) showed a significant association between the number of sessions and MBC changes (Table 2). The number of sessions was associated with a 40.2 mL loss in MBC [regression coefficient β: − 40.2, 95% confidence interval (CI): − 49.6, − 30.8]. The analysis of Model 2, in which Model 1 was adjusted to include the amount of time since the first electrocautery session at each subsequent session, showed a significant association between MBC changes and the number and duration of electrocautery sessions. MBC decreased by 55.5 mL (95% CI − 72.8, − 38.2) with each additional session, but this MBC loss was reversed by 10.9 mL (95% CI 1.91, 20.0) for each year that elapsed until the following session. Model 3, which was adjusted for clinical parameters, still showed a significant association between MBC changes and both the number of sessions (β: − 52.6 mL, 95% CI − 88.9, − 16.3) and the period between each session and the first session (β: 9.55 mL, 95% CI 3.60, 21.5). Furthermore, patients with low MBC (< 400 mL) at the first session had a higher risk of MBC loss with additional electrocautery sessions than those with normal MBC (≥ 400 mL) (Table 2). However, patient demographics, the extent of Hunner lesions, and symptom severity, including O’Leary and Sant Symptom and Problem indices (OSSI/OSPI), pain intensity, and urinary frequency, were not associated with MBC changes over time.

**Table 1:** Demographics of the study participants.

No. of patients (male/female)	118 (16/102)
Mean age at symptom onset (years)	62.8 ± 12.1 [30–86]^†^
Duration of illness (years from symptom onset to the first visit to our institution)	3.2 ± 4.0 [0–23]
Follow-up period (months from the first session to the last session)	38.5 ± 28.5 [5–134]
Number of surgeries	2.7 ± 1.2 [2–8]
OSSI	14.8 ± 3.9 [2–20]
OSPI	12.6 ± 3.4 [1–16]
Pain score^‡^	7.3 ± 2.3 [0–10]
Daytime urination frequency	17.5 ± 6.3 [7–36]
Nocturia frequency	4.4 ± 2.1 [0–12]
Average voided volume (mL)	118.9 ± 57.6 [31–300]
Maximum voided volume (mL)	181.1 ± 85.3 [50–420]
MBC at the first hydrodistension (mL)	460.4 ± 161.5 [70–1000]
Extent of Hunner lesions (%)^¶^	0.3 ± 0.19 [0.05–0.8]

MBC: maximum bladder capacity, OSSI/OSPI: O’Leary-Sant symptom index/O’Leary-Sant problem index.

^†^Mean ± SD [range].

^‡^Assessed using an 11-point pain intensity numerical rating scale from 0, indicating no pain, to 10, indicating the worst pain imaginable.

^¶^Defined as the relative bladder luminal surface area of Hunner lesions at surgery (Ref.^25^).

**Table 2:** Results of mixed-effect linear regression analyses for MBC against clinical parameters.

Clinical parameters	Model 1		Model 2		Model 3	
	β (95% CI)^†^	*P*-value	β (95% CI)	*P*-value	β (95% CI)	*P*-value
Number of surgeries	-40.2 (− 49.6, − 30.8)	< 0.001*	− 55.5 (− 72.8, − 38.2)	< 0.001*	− 52.6 (− 88.9, − 16.3)	< 0.001*
Time from the first surgery at each session (years)			10.9 (1.91, 20.0)	0.01*	9.55 (3.60, 21.5)	0.03*
Mean age at symptom onset (years)					− 1.92 (− 4.80, 0.963)	0.19
Sex (female)					− 9.30 (− 39.0, 20.4)	0.54
OSSI					5.28 (− 2.37, 12.9)	0.17
OSPI					− 1.64 (− 9.80, 6.53)	0.69
Pain scale‡					6.79 (− 2.90, 16.5)	0.17
Daytime urinary frequency					− 1.64 (− 7.12, 2.67)	0.37
Nocturia frequency					− 2.23 (− 15.7, 10.3)	0.68
Average voided volume (mL)					0.546 (− 0.154, 1.25)	0.12
Maximum voided volume (mL)					0.216 (− 0.240, 0.670)	0.35
MBC at the first hydrodistension < 400 (mL)					− 67.5 (− 37.6, − 95.2)	< 0.001*
Extent of Hunner lesions (%)^¶^					− 67.5 (− 216, 81.4)	0.36

^†^*β* regression coefficient, *CI* confidence interval (95%), *MBC* maximum bladder capacity, *OSSI/OSPI* O’Leary-Sant symptom index and problem index.

^‡^Assessed using an 11-point pain intensity numerical rating scale from 0, indicating no pain, to 10, indicating the worst pain imaginable.

^¶^Defined as the relative bladder luminal surface area of Hunner lesions at surgery (Ref.^25^).

*P < 0.05, statistically significant.

## Discussion

In the present study, we explored the relationship between the frequency of electrocautery of Hunner lesions and MBC over time using a mixed-effect linear regression model. There was a significant relationship between the number of electrocautery sessions and MBC loss (an approximately 50 mL reduction in MBC for each additional electrocautery session), and between the time from the first session to a subsequent session (an approximately 10 mL reduction in MBC loss for each year the subsequent electrocautery was postponed). We also found that low MBC (< 400 mL) at the first session was a prognostic marker for decreased MBC over additional sessions.

Endoscopic elimination of Hunner lesions, usually performed in combination with bladder hydrodistension, is a promising surgical treatment option for HIC^12,19–21^. However, HIC patients frequently need to undergo repeated surgeries due to the recurrence of Hunner lesions within a few years^12,15,22^. Repeated electrocautery of Hunner lesions may diminish the bladder capacity over time in HIC patients. With respect to this concern, previous studies have reported that multiple surgeries did not negatively affect bladder capacity over time^16–18^. Tomoe et al. reported that repeated electrocautery of Hunner lesions with hydrodistension did not diminish functional bladder capacity, but rather increased it^16^. Walker et al. reported that multiple sessions of bladder hydrodistension with concomitant fulguration of Hunner lesions did not significantly diminish MBC over time in 17 HIC patients^17^. Chennamsetty et al. compared the MBC at the initial and final electrocautery sessions in 51 HIC patients and reported that multiple sessions of electrocautery with bladder hydrodistension (mean, 2.98 ± 0.25 sessions, with a mean of 14.52 ± 1.34 months between sessions) did not significantly diminish MBC (mean difference: − 16.22 ± 20.72 mL, *p* = 0.437)^18^. The discrepancy between these studies and our present study may be due to the different methodology, such as a wide range of variations in study sample size or various statistical models that were used. In addition, evaluation criteria for bladder capacity also differed among studies; rather than evaluating MBC, some studies evaluated changes in functional bladder capacity, as measured by the maximum voided volume (MVV) or the average voided volume (AVV). However, functional bladder capacity (MVV) does not necessarily correlate with MBC, since it can be affected by lower urinary tract symptoms and pain. Indeed, in the present study, MVV and AVV did not significantly correlate with MBC over time. Given its objective nature, MBC at hydrodistension under general/spinal anesthesia is the preferred metric for assessing longitudinal changes in bladder anatomy.

This study also demonstrated that low MBC at hydrodistension may be a poor prognostic marker for bladder contraction. With respect to this, the duration of illness in subjects with low MBC was significantly longer than in those with normal MBC [low MBC (n = 43): mean duration, 4.2 ± 4.2 years; normal MBC (n = 75), 2.6 ± 3.8 years; *p* = 0.03], which might suggest that the subjects with low MBC had more advanced disease at the time of the first session than those with normal MBC. However, the number of sessions was still significantly associated with a decrease in MBC in subjects with normal MBC at the first session (Supplementary Fig. S1 and Table S2).

The strengths of this study include the large sample size (n = 118 HIC patients). To the best of our knowledge, the present study represents the largest sample set of HIC patients undergoing multiple sessions of electrocautery of Hunner lesions to be analyzed. Second, MBC changes were analyzed longitudinally using a mixed-effect linear regression model. Ordinary least square models or repeated measures ANOVA, which are frequently used for the analysis of longitudinal data, do not have the flexibility to determine the effects of multiple factors simultaneously and thus occasionally yield misleading results. Mixed-effect models have this flexibility and can address specific questions of clinical importance^23^.

The present study has several limitations. First, the retrospective nature of the study design may have biased the study results. Second, the decision of performing a repeat session was not made in a standardized manner and was determined by discussion with each patient. Third, surgeries were not performed by a single surgeon but by four experienced surgeons. Finally, the present results show a statistical relationship between multiple electrocautery sessions and MBC changes but do not indicate any cause-effect relationship. Discussion on any causal relationship will depend on further research clarifying the biological mechanisms of HIC progression.

In summary, the mixed-effect linear regression analyses performed here identified a statistically significant relationship between the frequency of electrocautery of Hunner lesions and MBC changes in HIC patients. In addition, low MBC at the first session is a poor prognostic marker for MBC loss over further sessions.

## Methods

### Ethics statement

The Institutional Review Board of the University of Tokyo approved this study, including the use of an opt-out methodology to obtain informed consent (approval no. 3124). Participants were informed about the study using generally accessible contact information and written informed consent was obtained from participants that chose to participate. All procedures followed appropriate guidelines.

### Participants and study design

A clinical data set of HIC patients who underwent multiple sessions of electrocautery of Hunner lesions with bladder hydrodistension from 2003 to 2020 was retrieved from a prospective, single-center clinical database of IC/BPS patients at our institution. Disease diagnoses and classifications were made according to American Urological Association and East Asian clinical guidelines for IC/BPS^4,24^. The database contained clinical information on the patients with IC/BPS, including the age of onset and the symptoms at the first visit; OSSI/OSPI scores; a pain intensity numerical rating on an 11-point scale (from 0, indicating no pain, to 10, indicating the worst pain ever); and frequency volume chart variables including daytime urinary frequency, nocturia frequency, AVV, and MVV. At surgery, Hunner lesions were carefully identified while the bladder was minimally filled with normal saline. Subsequently, the bladder was distended with normal saline to the maximum capacity at an intravesical pressure of 80 cm H_2_O for 3 min and then emptied. The amount of drained saline was considered the maximum bladder capacity (MBC). The presence and extent of Hunner lesions, MBC, and post-distension bleeding were documented as described previously^25^. All Hunner lesions were electrically resected or fulgurated completely. All surgeries were performed by one of four experienced surgeons (YA, NiA, NoA, and YH) under general or spinal anesthesia in accordance with standardized protocols at our institution.

### Statistical analysis

In this study, a mixed-effect linear regression model was used to assess potential MBC changes with repeated sessions, where the MBC at each surgery was nested in the patient. This model employed a fixed slope and random intercepts, which allowed for deviations of each patient’s intercept (Fig. 2). This linear regression model assumed the same (fixed) slope but allowed different (random) intercepts across different patients. First, the regression coefficient (β) and 95% confidence interval (CI) for MBC was estimated against the number of sessions (Model 1). Although an ordinary least squares regression model showed a similar pattern in a priori analysis (Table S1), we chose a mixed-effect model as the main analytic model because it allowed us to determine the effects of multiple factors on repeated measures flexibly, unlike a repeated measures analysis of variance (ANOVA)^23^.

Next, we controlled for potential confounding variables that may affect the primary explanatory variable (the number of sessions) and the independent variable (MBC). In Model 2, we adjusted for the period (years) from the first session to each subsequent session, since we hypothesized that the number and timing of the sessions could have different effects on MBC. In Model 3, the model was fully adjusted for additional clinical parameters, including the age of onset, sex, OSSI/OSPI scores, pain scale score, frequency of daytime urination and nocturia, AVV and MVV, the MBC at the first session (low MBC of < 400 mL or normal MBC ≥ 400 mL), and the extent of Hunner lesions (defined as the relative luminal surface area of Hunner lesions, see reference 20). The alpha was set to 0.05, and all *P*-values were two-sided. Data were analyzed using JMP software, version 14 (SAS Institute, Cary, NC, USA).

### Consent to participate

Participants were informed about the study using generally accessible contact information and written informed consent was obtained from participants that chose to participate.

### Consent for publication

All authors consent to the publication of the manuscript, should the article be accepted by the Editor-in-chief upon completion of the refereeing process. All participants consent to the publication of the study in a journal, Web site or other form of publication.

## Supplementary Information

Supplementary Figures.

Supplementary Tables.

## Data Availability

The data that support the findings of this study are available from the corresponding author upon reasonable request.

## Abbreviations

ANOVA: Analysis of variance
AVV: Average voided volume
HIC: Hunner type interstitial cystitis
IC/BPS: Interstitial cystitis/bladder pain syndrome
MBC: Maximum bladder capacity
MVV: Maximum voided volume
OSSI/OSPI: O’Leary and Sant Symptom and Problem Indices
