# Supplementary material for: Relationship between the frequency of electrocautery of Hunner lesions and changes in bladder capacity in patients with Hunner type interstitial cystitis
Source: Sci Rep. 2021 Jan 8;11:105. doi: 10.1038/s41598-020-80589-3 (PMC7794499; doi:10.1038/s41598-020-80589-3)
Supplement: Supplementary file 1 — Supplementary Figures. [file 41598_2020_80589_MOESM1_ESM.docx]

**Relationship between the frequency of electrocautery of Hunner lesions and changes in bladder capacity in patients with Hunner type interstitial cystitis**

Yoshiyuki Akiyama^1*^, Masayoshi Zaitsu^2^, Daiji Watanabe^1^, Itsuki Yoshimura^3^, Aya Niimi^4^, Akira Nomiya^5^, Yuta Yamada^1^, Yusuke Sato^1^, Masaki Nakamura^1^, Taketo Kawai^1^, Daisuke Yamada^1^, Motofumi Suzuki^1^, Haruki Kume^1^, Yukio Homma^6^

^1^Department of Urology, Graduate School of Medicine, The University of Tokyo, Tokyo, Japan

^2^Department of Public Health, Dokkyo Medical University School of Medicine, Tochigi, Japan

^3^Depratment of Urology, Teikyo University School of Medicine, Tokyo, Japan

^4^Department of Urology, New Tokyo Hospital, Matsudo, Chiba, Tokyo, Japan

^5^Department of Urology, National Center for Global Health and Medicine, Tokyo, Japan

^6^Japanese Red Cross Medical Center, Tokyo, Japan

**Supplementary Figure S1: A mixed-effect regression model for MBC and the number of surgeries in patients with normal MBC**


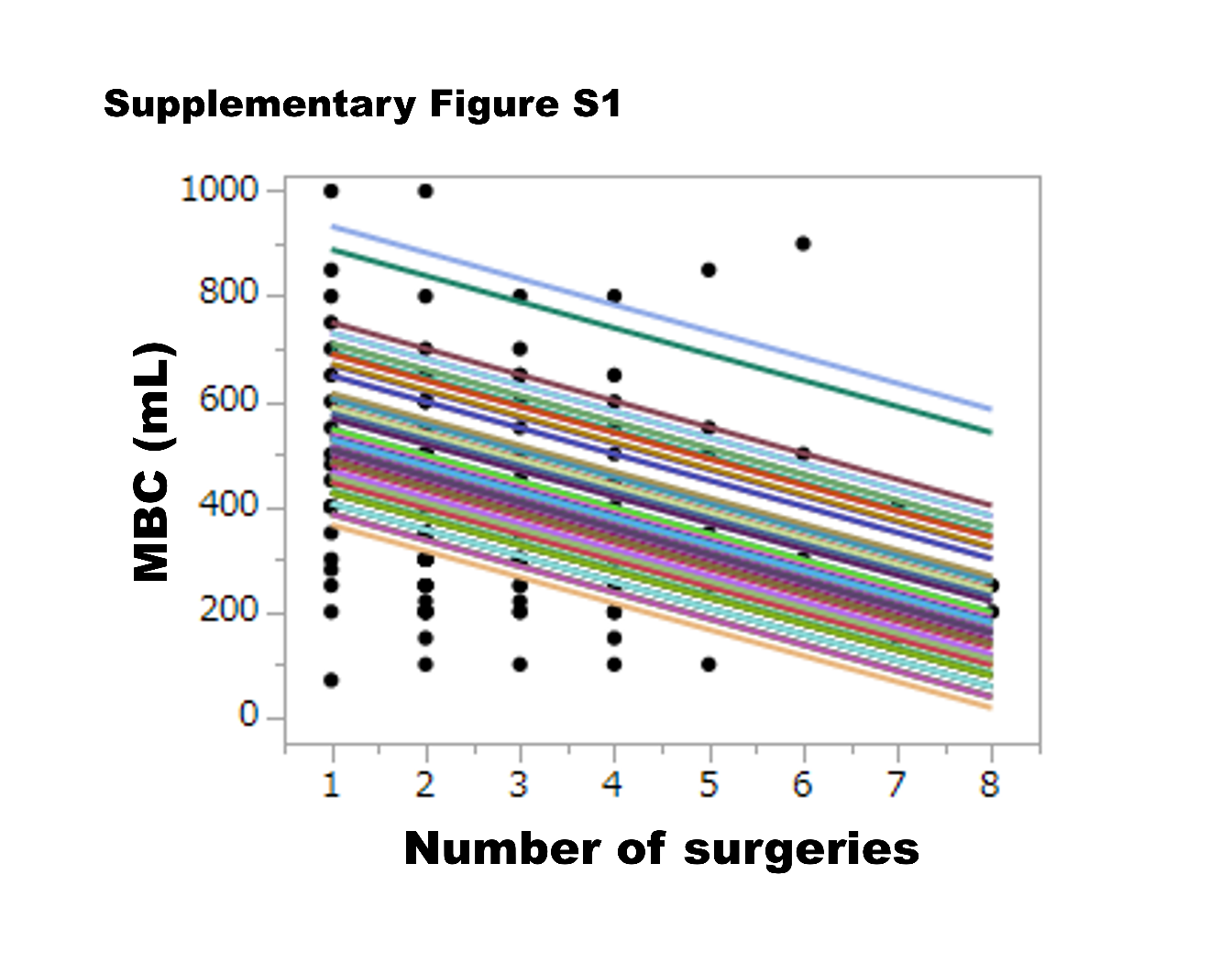


The dots represent individual MBC measurements over time. Each colored line depicts the fitted regression line for an individual patient, with a fixed slope and random intercepts.
