# Supplementary material for: Relationship between the frequency of electrocautery of Hunner lesions and changes in bladder capacity in patients with Hunner type interstitial cystitis
Source: Sci Rep. 2021 Jan 8;11:105. doi: 10.1038/s41598-020-80589-3 (PMC7794499; doi:10.1038/s41598-020-80589-3)
Supplement: Supplementary file 2 — Supplementary Tables. [file 41598_2020_80589_MOESM2_ESM.docx]

**Relationship between the frequency of electrocautery of Hunner lesions and changes in bladder capacity in patients with Hunner type interstitial cystitis**

Yoshiyuki Akiyama^1*^, Masayoshi Zaitsu^2^, Daiji Watanabe^1^, Itsuki Yoshimura^3^, Aya Niimi^4^, Akira Nomiya^5^, Yuta Yamada^1^, Yusuke Sato^1^, Masaki Nakamura^1^, Taketo Kawai^1^, Daisuke Yamada^1^, Motofumi Suzuki^1^, Haruki Kume^1^, Yukio Homma^6^

^1^Department of Urology, Graduate School of Medicine, The University of Tokyo, Tokyo, Japan

^2^Department of Public Health, Dokkyo Medical University School of Medicine, Tochigi, Japan

^3^Depratment of Urology, Teikyo University School of Medicine, Tokyo, Japan

^4^Department of Urology, New Tokyo Hospital, Matsudo, Chiba, Tokyo, Japan

^5^Department of Urology, National Center for Global Health and Medicine, Tokyo, Japan

^6^Japanese Red Cross Medical Center, Tokyo, Japan

Supplementary Table S1. Comparison of the results of an ordinal least squares model and a mixed-effect linear model

|  | Ordinary least squares model | |  | Mixed-effect linear model | |
| --- | --- | --- | --- | --- | --- |
|  | **β**† | ***P*-value** |  | **β** | ***P*-value** |
| **Model 1** |  |  |  |  |  |
| Number of surgeries | −20.1 | <0.001* |  | −40.2 | <0.001* |
| **Model 2** |  |  |  |  |  |
| Number of surgeries | −30.3 | <0.001* |  | −55.5 | <0.001* |
| Time from the first surgery at each session (years) | 7.75 | 0.02* |  | 10.9 | 0.01* |
| **Model 3** |  |  |  |  |  |
| Number of surgeries | −50.2 | <0.01* |  | −52.6 | <0.001* |
| Time from the first surgery at each session (years) | 7.10 | 0.41 |  | 9.55 | 0.03* |
| Mean age at symptom onset (years) | −2.13 | 0.06 |  | −1.92 | 0.19 |
| Sex (female) | −5.14 | 0.64 |  | −9.30 | 0.54 |
| OSSI | 4.77 | 0.09 |  | 5.28 | 0.17 |
| OSPI | −2.89 | 0.54 |  | −1.64 | 0.69 |
| Pain scale‡ | 8.21 | 0.21 |  | 6.79 | 0.17 |
| Daytime urinary frequency | −1.62 | 0.35 |  | −1.64 | 0.37 |
| Nocturia frequency | −5.14 | 0.28 |  | −2.23 | 0.68 |
| Average voided volume (mL) | 0.609 | 0.06 |  | 0.546 | 0.12 |
| Maximum voided volume (mL) | 0.176 | 0.42 |  | 0.216 | 0.35 |
| MBC at the first hydrodistension>400 (mL) | 64.2 | <0.001* |  | 67.5 | <0.001* |
| Extent of Hunner lesions (%)¶ | −73.4 | 0.24 |  | −67.5 | 0.36 |

†β: regression coefficient, MBC: maximum bladder capacity, OSSI/OSPI: O’Leary-Sant symptom index and problem index

‡Assessed using an 11-point pain intensity numerical rating scale from 0, indicating no pain, to 10, indicating worst ever

¶Defined as the relative bladder luminal surface area of Hunner lesions at surgery (ref. 25)

* *P*<0.05, statistically significant

Supplementary Table S2. Results of mixed-effect linear regression analyses for MBC against clinical parameters in patients with normal MBC (n = 75)

| **Clinical parameters** | **Model 1** | | **Model 2** | | **Model 3** | |
| --- | --- | --- | --- | --- | --- | --- |
|  | **β (95% CI)†** | ***P*-value** | **β (95% CI)** | ***P*-value** | **β (95% CI)** | ***P*-value** |
| Number of surgeries | -49.6 (−60.1, −39.1) | <0.001* | −62.1 (−81.1, −43.1) | <0.001* | −63.4 (−105, −21.7) | <0.01* |
| Time from the first surgery at each session (years) |  |  | 7.53 (−2.81, 17.9) | 0.15 | 3.10 (−16.2, 22.4) | 0.75 |
| Mean age at symptom onset (years) |  |  |  |  | −3.80 (−8.85, 1.30) | 0.13 |
| Sex (female) |  |  |  |  | −21.8 (−108, 64.9) | 0.60 |
| OSSI |  |  |  |  | 15.5 (−5.20, 36.2) | 0.13 |
| OSPI |  |  |  |  | −7.55 (−27.2, 12.1) | 0.43 |
| Pain scale‡ |  |  |  |  | 3.32 (−17.7, 24.3) | 0.74 |
| Daytime urination frequency |  |  |  |  | −11.2 (−25.2, 2.73) | 0.11 |
| Nocturia frequency |  |  |  |  | 8.20 (−28.8, 45.1) | 0.65 |
| Average voided volume (mL) |  |  |  |  | 0.741 (−0.691, 2.17) | 0.29 |
| Maximum voided volume (mL) |  |  |  |  | 0.016 (−0.934, 0.965) | 0.97 |
| Extent of Hunner lesions (%)¶ |  |  |  |  | 106 (−237, 450) | 0.52 |

CI: confidence interval (95%), MBC: maximum bladder capacity, OSSI/OSPI: O’Leary-Sant symptom index and problem index

†β: regression coefficient

‡Assessed using an 11-point pain intensity numerical rating scale from 0, indicating no pain, to 10, indicating worst pain ever

¶Defined as the relative bladder luminal surface area of Hunner lesions at surgery (ref. 25)

* *P*<0.05, statistically significant
